# Entwicklung des Alkoholkonsums zu Beginn und während der ersten Wellen der SARS-CoV-2-Pandemie: Ergebnisse einer systematischen Literaturrecherche

**DOI:** 10.1007/s10049-022-01031-x

**Published:** 2022-04-29

**Authors:** Beatrice Thielmann, Irina Böckelmann, Heiko Schumann

**Affiliations:** Bereich Arbeitsmedizin, Medizinische Fakultät, Otto-von-Guericke-Universität Magdeburg, Leipziger Str. 44, 39120 Magdeburg, Deutschland

**Keywords:** Spirituose, Trinkverhalten, Pandemie, Gesundheitliche Risiken, Prävention, Spirit drink, Drinking behavior, Pandemic, Health risk behaviors, Prevention

## Abstract

**Hintergrund und Ziel der Arbeit:**

Es ist bekannt, dass der Alkoholkonsum und -missbrauch sowie alkoholinduzierte Probleme in wirtschaftlich schwierigen Zeiten zunehmen – was frühere SARS-Studien belegen. Das Review untersucht weltweite Veränderungen des Alkoholkonsums unter dem Einfluss der aktuellen SARS-CoV-2-Pandemie.

**Material und Methoden:**

Es wurden die Datenbanken PubMed, Ovid, Cochrane Library, Scopus, PsycINFO und Web of Science mit Stichtag 11.01.2022 verwendet. Es fand sich eine initiale Trefferzahl von 791 Publikationen. Nach Lesen von Titel und Abstract kamen noch 62 Texte infrage. Nach Sichtung des Volltexts wurden 40 Studien in dieses Review einbezogen.

**Ergebnisse:**

Studienergebnisse lagen aus Nord- und Südamerika, Europa, Asien und Ozeanien vor. Es zeigte sich sowohl ein Anstieg als auch eine Reduktion des Alkoholkonsums. Studien, die über mehrere Wellen der Pandemie untersuchten, fanden einen Alkoholanstieg in Relation zur Dauer der Pandemie. Das Binge-Drinking spielte dabei eine große Rolle. Es gab sehr große regionale Unterschiede beim Anstieg des Alkoholkonsums: von ca. 10 % der Befragten auf > 45 %. In den meisten Studien war der Alkoholkonsum bei 40–50 % der Befragten etwa gleich und bei 30–40 % verringerte er sich.

**Diskussion:**

Weitere Studienverläufe unter anhaltender Pandemie sind wichtig. Da die untersuchte Bevölkerung überwiegend im berufstätigen Alter war, erscheinen betriebliche Präventionsmaßnahmen bei erhöhter Stressbelastung für einen Teil der Befragten mit erhöhtem Alkoholkonsum als sinnvoll.

**Zusatzmaterial online:**

Die Online-Version dieses Beitrags (10.1007/s10049-022-01031-x) enthält eine Übersicht der Artikel zum Thema Alkoholkonsum in Pandemiezeiten.

## Abstract (en)

**Background and objective:**

It is well known that alcohol consumption and abuse, as well as alcohol-induced problems, increase during difficult economic times. Previous studies showed increased alcohol consumption in the 2003 SARS outbreak in China. The review examines global changes in alcohol consumption under current SARS-CoV‑2 (severe acute respiratory syndrome coronavirus 2) pandemic.

**Materials and methods:**

The databases PubMed, Ovid, Cochrane Library, Scopus, PsycINFO, and Web of Science with a cut-off date of 11 January 2022 were used. An initial hit count of 791 publications was found. After reading the title and abstract, 62 texts were still eligible. After reviewing the full text, 40 studies were included in this review.

**Results:**

Study results were available from North and South America, Europe, Asia and Oceania. Increases as well as reductions in alcohol consumption were shown. Studies examining multiple waves of the pandemic found that alcohol consumption increased with duration of the pandemic. Binge drinking played a large role in this. There were very large regional differences in the increase in alcohol consumption: from about 10% of respondents to > 45%. In most studies, alcohol consumption was about the same for 40–50% of respondents and decreased for 30–40%.

**Conclusions:**

Further study follow-ups under the continuing pandemic are relevant. Since the populations studied were predominantly of working age, occupational prevention measures of elevated stress levels for some of the respondents with increased alcohol consumption seem reasonable.

Unter der aktuellen SARS-CoV-2-Pandemie (severe acute respiratory syndrome coronavirus type 2) haben sich das öffentliche Leben und der Alltag, inklusive Berufsalltag, verändert. Erste Daten zu indirekten Auswirkungen der Pandemie auf die psychische Gesundheit, wie bspw. den Alkoholkonsum, liegen jetzt vor. Erste Daten weisen auf einen Trend des erhöhten Rauschtrinkens aufgrund des erlebten Stresses durch bspw. Ausgangsbeschränkungen hin. Diese Übersicht stellt den Verlauf des Alkoholkonsums zu Beginn und während der ersten Wellen der Pandemie dar.

## Hintergrund

Beschäftige vieler Berufsgruppen unterliegen immer mehr hohen psychosozialen Belastungen, die zu gesundheitlichen Folgen führen [6, 26]. Diese Belastungen werden während der SARS-CoV-2-Pandemie noch verstärkt. So zeigt sich eine Verdichtung der Arbeit auf einigen Stationen in Krankenhäusern. Angst um persönliche Sicherheit und Gesundheitsschutz am Arbeitsplatz, wie sie in Gesundheits‑/Pflegeberufen in der Pandemie vorkommt, verstärkt die ohnehin hohe psychische Belastung in diesen Berufsgruppen [3, 32, 50]. Angst vor Kurzarbeit und plötzlicher Arbeitslosigkeit bei Beschäftigten vieler Betriebe, Existenz- und Armutsängste der Selbstständigen aufgrund einer weggebrochenen Geschäftsgrundlage und dazu noch die allgemeinen Maßnahmen des Lockdowns wie Ausgangssperren, „social distancing“ und Isolation sind einschneidend. Hinzu kommen Mehrfachbelastungen in den Familien durch die fehlende Kinderbetreuung, Homeschooling und/oder Homeoffice des Partners [50]. Das alles kann zu Überbeanspruchungen und Überreaktionen (Stichwort: Gewalt in der Familie) führen.

Eine falsch gewählte Bewältigungsstrategie von Stress kann ein erhöhter Alkoholkonsum sein, was zunächst zu einer subjektiven Veränderung der Belastung führt [47, 49] und langfristig mit negativen gesundheitlichen Folgen in Verbindung steht [42].

Stress kann ein Risikofaktor für den problematischen Alkoholkonsum sein [62]; beides steht häufig in Verbindung [9]. Deutschland gehört zu den Alkoholhochkonsumländern [63]. 14,2 % der 18- bis 64-Jährigen, d. h. der erwerbsfähigen Bevölkerung Deutschlands, weisen einen riskanten Alkoholkonsum auf, wovon 3,4 % abhängig sind [45]. Ein riskanter Konsum wird bei der Aufnahme von mehr als 12 g Reinalkohol pro Tag in den letzten 30 Tagen bei Frauen und 24 g bei Männern angesehen [8]. Daten des Epidemiologischen Suchtsurveys ergaben, dass 71,6 % der Befragten und somit 36,9 Mio. Menschen in Deutschland Alkohol in den letzten 30 Tagen vor der Befragung konsumierten [4]. Die indirekten Kosten, wie Mortalitätsverluste, Arbeitsunfähigkeit, Rehabilitation, Frühberentung und Produktionsausfälle, machen ca. 16,6 Mrd. € aus [8]. Sachschäden alkoholbedingter Arbeitsunfälle belaufen sich auf über 1 Mrd. € [8]. Für das Individuum relevanter sind die durch schädlichen und abhängigen Alkoholkonsum entstehenden erheblichen psychosozialen Auswirkungen. Sie bedeuten Verlust an Lebensqualität der betroffenen Menschen sowie deren Angehöriger [63]. Somatisch können alle Organe infolge einer Alkoholabhängigkeit geschädigt sein; im Vordergrund stehen dabei die alkoholische Leberkrankheit und chronische Pankreatitis sowie neuropsychiatrische Schäden wie Hirnatrophie, Enzephalopathie und Alkoholpsychose [27].

Es gibt weit verbreitete Besorgnis darüber, dass die COVID-19-Pandemie ein hohes Risiko für den Alkoholkonsum unter stark trinkenden Bevölkerungsgruppen aufweist. Daher war das Ziel der Literaturrecherche, die Entwicklungen des Alkoholkonsums in Pandemiezeiten weltweit zu beurteilen. Dabei sollten entweder Veränderungen des Konsumverhaltens während der Pandemie bestimmt oder auf Studien vor Pandemiezeiten zurückgegriffen und verglichen werden. Aussagen zu Berufsgruppen sollten ebenfalls erfasst werden, wobei das Interesse des Reviews auf Gesundheitspersonal und/oder Rettungsdienstpersonal lag.

## Methodik

Es erfolgte eine systematische Literaturrecherche in den Datenbanken PubMed, Ovid, Cochrane Library, Scopus, PsycINFO und Web of Science (Deadline: 11. Januar 2022). Als Suchbegriffe wurden („alcoholism“ ODER „alcohol“) UND („pandemic“ ODER „corona“ ODER „SARS-CoV-2“ ODER „Covid 19“) definiert. Einschlusskriterien waren Studien, die den Alkoholkonsum zu verschiedenen Zeiten der Pandemie, im Vergleich zu Zeiten vor der Pandemie oder Veränderungen des Alkoholkonsums während der Pandemie abfragten. Dabei wurden nur Research-Artikel gesichtet. Ausschlusskriterien waren somit Darstellung von Studienergebnissen in Form von Editorials, „letter to editor“, Fallbeschreibungen oder Ähnliches, Studien zum Alkoholkonsum bei Jugendlichen unter 18 Jahren und Studien, die den Alkoholkonsumverlauf während der Pandemie bei Alkoholabhängigen oder Abstinenten untersuchten. Die gefundenen Artikel wurden in den Referenzmanager Citavi 6 (Swiss Academic Software, Wädenswil, Schweiz) eingefügt und Duplikate entfernt. Die Autoren BT und HS überprüften unabhängig voneinander Titel und Abstracts nach dem Thema. Es wurden englisch- und deutschsprachige Arbeiten berücksichtigt. Der Zeitraum wurde ab 2020 definiert, da die Pandemie 03/2020 durch die WHO erklärt wurde [65]. Die genannten Autoren überprüften unabhängig voneinander den Volltext dieser Artikel. Unstimmigkeiten wurden durch Diskussion mit einem dritten Gutachter (IB) geklärt.

## Ergebnisse

### Allgemeine Auswertungen

**Figure 1:**
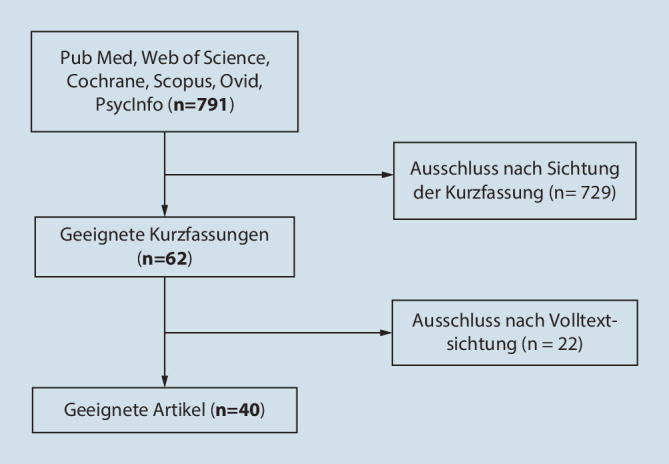

Die erste Suche ergab nach Ausschluss von Dubletten 791 Datensätze. Nach Prüfung von Titel und Kurzfassung kamen 40 Veröffentlichungen infrage [1, 2, 5, 10–16, 19–22, 24, 25, 28–31, 33, 35–41, 43, 44, 46, 48, 51, 53–57, 61, 64]. Eine Übersicht über die eingeschlossenen Studien und Vorgehensweisen zeigt Abb. 1. Eine tabellarische Übersicht über die untersuchten Artikel finden Sie im Online-Zusatzmaterial.

Der Erhebungszeitraum umfasste bei allen Publikationen das Frühjahr bis zum Herbst 2020 und betraf überwiegend die erste und zweite Welle der SARS-CoV-2-Pandemie. Keine der eingeschlossenen Studien bot Daten aus dem Jahr 2021. Nur sieben Arbeiten boten einen Vorher-nachher-Vergleich an [2, 10, 16, 31, 40, 43, 64] mit Daten vor der Pandemie. Sechs Studien befragten zu mehreren Zeitpunkten während der Pandemie [2, 12, 15, 16, 36, 39].

Eine Studie realisierte Beobachtungen über einen Zeitraum von sechs Monaten [10], die meisten anderen Studien beobachteten sieben Tage bis acht Wochen. Der Alcohol Use Disorder Identification Test (AUDIT), inklusive einer Subversion (AUDIT-C), war der häufigste verwendete standardisierte Fragebogen in elf Studien [1, 11, 13, 16, 25, 28, 29, 33, 37, 43, 55], ansonsten kamen nichtstandardisierte Fragebögen zur Anwendung. Dabei wurde der Alkoholkonsum im Vergleich zur Vor-Coronapandemie-Zeit erfragt. Eine Studie bestimmte Ethylglucuronid in Haaren als Nachweis von Alkoholkonsum [2].

### Verteilung der Studienpopulation und Herkunft der Studien

Nur bei vier Studien war die Mehrheit der Befragten Männer [1, 10, 43, 61]. Bei zwei Studien waren jeweils die Hälfte Frauen und Männer [46, 51], ansonsten waren Frauen die Mehrheit der Befragten. Eine Studie untersuchte nur Frauen [53]. Drei Studien machten keine Angaben zum Geschlecht [2, 14, 15] und 8 Studien keine genaueren Angaben zum Alter [2, 5, 16, 21, 25, 36, 37, 41]. In der Mehrheit der Studien zählten die Studienteilnehmer zur allgemeinen, nicht näher definierten Bevölkerung. Bei drei Studien waren Studenten [21, 25, 31], bei zwei Studien ein großer Anteil Gesundheitspersonal [29, 41] und in einer Studie Online-Spieler [46] eine ausgewählte Gruppe der Befragten.

Die Studien kamen aus Nordamerika [5, 13, 15, 16, 19, 29, 31, 33, 39–42, 44, 46, 51, 55, 61], Mittel‑/Südamerika [11, 21, 24], Europa [2, 10, 11, 14, 22, 25, 35, 37, 38, 43, 53, 54, 57, 64], Asien [1, 12, 28, 36, 48, 56] und Ozeanien [20, 30].

Der größte Teil der Studien nutzte auch psychologische Fragestellungen, überwiegend zu Angst und Depression, die hier nicht berücksichtigt wurden.

Prinzipiell zeigte sich bei auffälligen psychischen Werten für Angst und Depression ein erhöhter Alkoholkonsum im Vergleich zu Befragten ohne psychische Störungen. Nur zwei Studien untersuchten, ob eine COVID-Erkrankung vorlag [21, 44].

### Daten zum Alkoholkonsum – Konsummuster und -mengen

Es zeigte sich sowohl ein Anstieg als auch eine Reduktion des Alkoholkonsums, wobei Studien über mehrere Wellen der Pandemie einen Alkoholanstieg mit Dauer der Pandemie verzeichneten [2, 12, 16, 36, 39, 55, 64]. Das Binge-Drinking spielte dabei eine große Rolle [2, 10, 12, 61, 64].

Es gab sehr große regionale Unterschiede beim Anstieg des Alkoholkonsums: von ca. 10 % der Befragten [19, 37] auf > 45 % [30, 44, 55] in Neuseeland und den USA. In den meisten Studien war der Alkoholkonsum bei 40–50 % der Befragten etwa gleich und bei 30–40 % nahm dieser sogar ab [14, 28, 31, 57].

Nur wenige Studien machten Aussagen zur Prävalenz des Alkoholkonsums in der Bevölkerung. Diese wurde mit 45,6 % in Brasilien [11], 30,8 % in Spanien [11] und 9 % in Indonesien [28] angegeben.

Die Betrachtung des AUDIT-Scores ergab einen risikoarmen Alkoholkonsum [16, 25, 28]. Der AUDIT-C-Score wies bei Frauen ≥ 3 und bei Männern ≥ 4 Punkten auf ein risikoreicheres Trinken, was in einigen Studien zu beobachten war [13, 29, 37]. In der Studie von Boschuetz et al. war dies für Frauen signifikant [13]. Eine weitere Studie fand einen signifikant höheren Alkoholkonsum bei Frauen [14], ansonsten tranken signifikant mehr Männer als Frauen Alkohol [1, 12, 28, 36, 44]. Der Alkoholkonsum der Jüngeren war höher als der der Älteren [14, 25, 35, 43] – es war nur eine Studie zu finden, in der Ältere mehr tranken als Jüngere [28].

Unter COVID-Erkrankten lag der Anstieg des Alkoholkonsums bei 47,2 % der Betroffenen [44] bzw. 70,6 % (Vergleich zu Nichterkrankten 15,3 %; [21]).

Eine große europäische Studie mit > 35.000 Befragten fand zwar einen Rückgang des Alkoholkonsums in der Europäischen Union, aufgrund weniger Möglichkeiten des Rauschtrinkens, jedoch nahm der Gesamtkonsum in Deutschland und im United Kingdom im Vergleich zu anderen EU-Ländern zu [37].

Die Studie von Suffoletto et al. [55] untersuchte 50 Personen, die wegen Alkoholkonsum in die Notaufnahme eingeliefert wurden – Bei 44 % davon wurde ein gefährlicher Konsum in der ersten Woche des Lockdowns beschrieben. Es wurden staatliche Interventionen durchgeführt (z. B. Aufklärungsgespräche), die einen Abfall des gefährlichen Konsums nach der zweiten Woche auf 29 % erbrachten. Jedoch war dieser Effekt mit anhaltendem Lockdown nicht mehr zu beobachten. Der gefährliche Alkoholkonsum stieg sogar auf 65 % der Befragten an. Die Autoren schlussfolgerten, dass staatliche Maßnahmen nicht halfen. Eine weitere Studie mit einem Beobachtungszeitraum über 6 Monate zeigte einen Alkoholkonsumanstieg von 3,9 auf 17,4 % in Gruppen mit gefährlichem, schädigendem oder abhängigem Konsum [33]. Die Studie von Ahmed et al. [1] bot einen Anstieg des riskanten Alkoholkonsums um 29,1 % und des gefährlichen Konsums um 9,5 %. Nur 1,6 % von 1074 Befragten hatten bereits eine Alkoholabhängigkeit. Auch in der Studie von Alladio et al. [2] war zunächst eine Reduktion des Alkoholkonsums, dann jedoch wieder ein Anstieg des exzessiven Alkoholkonsums von 7 auf 9 % zu verzeichnen. Obwohl auch die Studie von Benschop et al. [10] eine Abnahme des Alkoholkonsums nachwies, nahm die Tages- und Wochenmenge an konsumiertem Alkohol zu. Weitere Studien verzeichneten entweder die Zunahme des Rauschtrinkens [12, 61, 64] oder der Trinktage im Verlauf der Pandemie [37, 39, 41, 51, 61]. Nur eine Studie zeigte eine Reduktion des starken oder der Anzahl der Tage des Alkoholkonsums [40]. Eine Studie berichtete, dass zwar Alkoholkonsum in geselliger Runde von 77,5 auf 65,0 % abnahm, aber das Online-Trinken von 6,3 auf 10,4 % zunahm [24].

Eine weitere Studie untersuchte medizinisches Personal in Italien. Dabei ergab sich eine Alkoholkonsumstörung bei 42,6 % der Befragten [29]. Die Studie von Mongeau-Pérusse et al. [41] fand heraus, dass Gesundheitspersonal zwar weniger hochprozentige Alkoholprodukte konsumierte, jedoch die Tage des täglichen Alkoholkonsums signifikant zunahmen.

## Diskussion

Dieses Review zeigt, dass der veränderte Alkoholkonsum eine Folge der pandemiebedingten Maßnahmen und der Zunahme psychischer Belastungen (Angst vor Verlust persönlicher und familiärer Sicherheit, „social distancing“, Mehrfachbelastungen in den Familien durch die fehlende Kinderbetreuung, Homeschooling usw.) sein könnte. Der Hauptteil der Befragten gab einen unveränderten Alkoholkonsum an. Es ist jedoch anzumerken, dass die Studien sich überwiegend auf die erste und zweite Welle konzentrierten. Studienergebnisse aus 2021 liegen noch nicht vor.

Anpassungen des Alkoholkonsums sind veränderte Alkoholkonsummuster wie bspw. Binge-Drinking oder Online-Trinken. Einige Studien belegen einen zunehmenden Alkoholkonsum mit anhaltender Dauer der Lockdown-Maßnahmen. Dabei muss beachtet werden, dass die Möglichkeiten von Rauschtrinken durch Pandemiemaßnahmen verringert, diese jedoch im Verlauf der weiteren Pandemie nicht mehr so streng waren. Hier könnten aber auch Unterschiede in Altersgruppen vermutet werden. Als Limitation dieser Literaturrecherche muss angemerkt werden, dass die Suchbegriffe wie „alcohol use“ oder „alcohol consumption“ ebenfalls geeignet gewesen wären, jedoch nicht berücksichtigt wurden.

Bereits vor der Pandemie konsumierten ca. 23 % der Arbeitnehmer regelmäßig Alkohol, davon 8 % Alkohol am Arbeitsplatz. Bei etwa 25 % der Betriebsunfälle war Alkohol im Spiel [7, 18]. Es werden enorme volkswirtschaftliche Kosten durch Alkohol verursacht [7, 18]. Obwohl die Studienergebnisse überwiegend die Allgemeinbevölkerung betrafen, können diese teilweise auf den Alltag des Arbeitnehmers bezogen werden, da der Hauptteil der Befragten im berufstätigen Alter war. Daher spielen geeignete betriebliche präventive Maßnahmen (nicht nur im Kontext des Alkoholkonsums, sondern auch hinsichtlich der psychischen Belastungen) eine Rolle, die vor allem den Mitarbeitenden zugutekommen, die ein Risiko für erhöhten Alkoholkonsum aufweisen. Eine Studie über Alkoholmissbrauchs‑/Abhängigkeitssymptome von Gesundheitspersonal drei Jahre nach dem SARS-Ausbruch in China 2003 fand einen positiven Zusammenhang mit der Tatsache, dass Betroffene unter Quarantäne gestellt wurden oder mit SARS-Patienten bzw. auf SARS-Stationen arbeiteten [66]. Die Autoren kamen zu der Schlussfolgerung, dass nicht nur Katastrophenexpositionen, sondern auch eine Exposition gegenüber dem Ausbruch einer schweren Infektionskrankheit zu einer posttraumatischen Belastungsstörung oder auch zu anderen psychiatrischen Erkrankungen wie Alkoholmissbrauch/-abhängigkeit führen kann [66].

Im Zusammenhang mit den zusätzlichen psychischen Belastungen während der Pandemie sind eine umfassende Information, eine Stärkung der Resilienz durch Tagesstrukturierung und eine frühzeitige betriebliche Prävention zur Reduzierung von berufsbedingtem Stress empfehlenswert und unabdingbar [60]. Ein erhöhter Alkoholkonsum als Coping-Mechanismus bei Stress ist die falsche Wahl. Eine Aufklärung zu möglichen Folgen des erhöhten Alkoholkonsums ist im Rahmen einer Suchtprävention notwendig [59]. Bewegungserhalt und gesunde Ernährung als gesundheitsförderliche Maßnahmen können ergänzend angeboten werden [58].

Im Rahmen einer Interventionslängsschnittstudie konnte eine Erfolgsquote von 50 % bezüglich Abstinenz und Reduktion auf harmloses Trinken erzielt werden [17]. Auch Führungskräfte sind in der Verantwortung und sollten eine betriebliche Suchtprävention implementieren; sie haben eine Fürsorgepflicht [18, 23]. Diese Aufgabe könnten der Betriebsarzt und sein Präventionsteam übernehmen, z. B. in Form von Schulungen, Beratungen in konkreten Problemfällen, Kontaktaufnahme mit Hausarzt, Familie, Behandlungseinrichtung und Therapievermittlung [18]. Ein Review belegte eine begrenzte Evidenz für eine geringe, aber wahrscheinlich relevante Reduktion des Stressniveaus durch personenzentrierte, personennahe und organisatorische Interventionen bei Beschäftigten [63]. Organisatorische Interventionen (z. B. Unterstützung, Kommunikation, flexible und familienfreundliche Arbeitszeiten) sollten sich auf die Reduzierung spezifischer Stressoren konzentrieren, d. h., es wird eine Evaluation bestehender Belastungen empfohlen, um diesen entgegenzuwirken [52]. Diesbezüglich besteht sicherlich auch ein hohes Forschungspotenzial. Inhomogene Evidenz soll natürlich auch nicht heißen, dass berufsbedingter Stress allein auf den Schultern der Mitarbeitenden zu tragen ist.

Auch Erkenntnisse des Kompetenznetzes Public Health zu COVID-19 aus Fachgesellschaften und Verbänden zur Abschätzung der psychosozialen Folgen von Isolations- und Quarantänemaßnahmen und deren Vorschläge zu den möglichen Lösungsansätzen sollen in die Arbeit der Präventionsteams einfließen [34]. Im Hinblick auf die Gefährdung bestimmter Personen, u. a. mit psychischen Vorerkrankungen wie Angststörungen und Depressionen, sollen die psychotherapeutischen Interventionen angedacht werden.

## Fazit für die Praxis

- Pandemiebedingt ist eine beinahe weltweite Zunahme des Alkoholkonsums zu beobachten, die zu gesundheitsgefährdenden Folgen führen kann.
- Die Zunahme des Alkoholkonsums ist durch veränderte Trinkmuster wie bspw. Rauschtrinken verursacht.
- Frühere SARS-Studien zeigten, dass die Exposition gegenüber Infektionskrankheiten zu erhöhtem Alkoholkonsum führt. Ein erhöhter Alkoholkonsum ist bei auffälligen psychischen Werten für Angst und Depression zu verzeichnen (hier nicht dargestellt).
- Zusätzlich besteht ein Bedarf an mehr Forschung über die Wechselwirkung von Alkoholkonsumverhalten und zusätzlichen Belastungen in der SARS-CoV-2-Pandemie, um ein besseres Verständnis für die potenziellen langfristigen Auswirkungen des Lockdowns auf die Gesundheit zu erhalten und spezifische Präventionsprogramme für die gefährdeten Beschäftigten zu entwickeln.
- Betriebliche Suchtpräventionsprogramme sollten implementiert bzw. optimiert werden, wobei noch Forschungspotenzial besteht.
- Kurse zur Stressreduzierung, Erarbeitung der positiven Stressbewältigungsstrategien und Stärkung der Resilienz sollten angeboten werden.

## Supplementary Information




