# Supplementary material for: Drinking behavior at the beginning and during the SARS-CoV-2 pandemic: results of a literature review
Source: Notf Rett Med. 2022 Apr 29:1–7. [Article in German] Online ahead of print. doi: 10.1007/s10049-022-01031-x (PMC9051819; doi:10.1007/s10049-022-01031-x)
Supplement: Supplementary file 1 [file 10049_2022_1031_MOESM1_ESM.pdf]

Zusatzmaterial zum Beitrag „Entwicklung des Alkoholkonsums zu Beginn und während der ersten Wellen der SARS-CoV-2-Pandemie. Ergebnisse einer systematischen Literaturrecherche“ von Thielmann B, Böckelmann I, Schumann H (2022) in *Notfall+Rettungsmedizin.*
Beitrag und Zusatzmaterial stehen Ihnen auf www.springermedizin.de zur Verfügung. Bitte geben Sie dort den Beitragstitel in die Suche ein.

Tabelle: Übersicht der Artikel zum Thema Alkoholkonsum in Pandemiezeiten

**Autoren[t]**	**Land**	**Anzahl N**	**Verteilung (Frauen♀/ Männer♂),**	**Alter (Jahren)**	**Zeitraum**	**Fragebogen Alkohol-Konsum**	**Prävalenz oder Konsum des Alkoholkonsum (A.)**
**Originalarbeiten**							
**Ahmed, 2020 [1]**	China	1.074	46,8% ♀, 53,2% ♂	14-68, 54±11,13	n. b.	AUDIT	↑ riskanter A. bei 29,1% und gefährlicher A. bei 9,5% 1,6% Abhängigkeit. ♂:♀ 6:1
**Alladio, 2021 [2]**	Italien	>1.000 pro Erhebung	n. b.	n. .b.	2019 05/20 07/20	Haaranalyse Ethylglucu-ronide (EtG).	05/19->05/20: geringer A. bei 60->79%, moderater A. bei 28->14%, exzessiver A. 12->7%; 05/20->07/20: geringer A. 79->79%, moderater A. 14->13%, exzessiver A. 7->9%
**Avery, 2020 [5]**	USA	3.989	69,2% ♀, 30,8% ♂	n. b.	03/20 - 05/20	n. s. F.	↑ A. bei 14,3% der Befragten; bei Stress Anstieg A. OR 1,37 (p<0.001); bei Angst OR 1,42 (p<0.001).
**Benschop, 2021 [10]**	Niederlande	5.176	48,9%,♀, 50,6% ♂	46,5% 18-24	2019 05/20-10/20	n. s. F.	↑ Tages- und Wochenmenge: 4,3->4,8*** 20,8->22,3***<br>↑ bei 32,1%, ↔ bei 21,2%, ↓ bei 29,1% Ø bei 11,3%
**Boni de, 2021 [11]**	Brasilien, Spanien	N=22.785, Brasilien 19.256, Spanien 3.529	68,9% ♀, 31,1% ♂	44,3% > 41	04/20-05/20	AUDIT-C	Prävalenz 43,3%<br>(45,6% Brasilien, 30,8% in Spanien)

**Bonny-Noach, 2021 [12]**	Israel	N= 750,<br>n1=427,<br>n2=323	72,8% ♀, 27,2% ♂	29,8±11,8	03-05/20<br>10-11/20	n. s. F.	♂>♀*** (p<0,001). 2>1 Sperrungen*<br>↑ Binge-Drinking***
**Boschuetz, 2020 [13]**	USA	407	83,5% ♀, 16,3% ♂,	ab 18	bis 04/20	AUDIT-C	Score 3 → 4. ♀ (*) bei Nichtabhängigen
**Bramness, 2021 [14]**	Norwegen	1.200		>18	06/20-<br>07/20	n. s. F.	↑ bei 13,3%; ♀>♂***, ↔ bei 56,8%, ↓ bei 29,9%; jüngere>mittlere>ältere***
**Charles, 2021 [16]**	USA	385	87,4% ♀, 11,9% ♂<br>Studenten		09-11/19<br>04/20<br>10-11/20	AUDIT	Score: t1: 3,29, t2: 4,14 und t3: 3,11
**Carpasso, 2021 [15]**	USA	10.780	n. b.	>18	03/20 und<br>04/20	n. s. F.	↑ bei 29%, ↔ bei 51,2% ,↓ bei 19,8%
**Eastman, 2021 [19]**	USA	6.548	53,5% ♀, 46,5% ♂	>55,<br>67,7±0,2;	03-04/20	n. s. F.	↑ bei 10,9%, ↔ bei 37,8%, ↓ bei 9,7%
**Every-Palmer et al., 2020 [20]**	Neuseeland	2.010	52,9% ♀, 46,8% ♂	18-90,<br>Ø 45	bis 04/20	n. s. F.	↑ bei 22,0%, ↔ bei 59,1%, ↓ bei 18,9%
**Fernandez, 2021 [21]**	Brasilien	1.050	70,6 % ♀, 29,4 % ♂<br>Studenten		07/20	n. s. F.	5,5% mit Covid-Erk.<br>davon 70,6% A. (Vgl. nicht erk. 15,3% A.); ♂>♀
**Ferrante, 2020 [22]**	Italien	7.847	71,3% ♀, 28,7% ♂	48,6±13,9	bis 06/20	n. s. F.	↑ bei 17,3%, 23% ♂ (*), 15,1% ♀
**Garcia-Cerde, 2021 [24]**	Lateinamerika, Karibik (33 Länder)	12.328	65,9% ♀, 34,1% ♂	38,1±12,8	05-06/20	n. s. F.	↓ 77,5%->65,0%,<br>Online-Trinken ↑ 6,3±16,6 -> 10,4±22,6%
**Gavurova, 2021 [25]**	Tschechien, Slowakei	CZ: 1.422<br>SK: 1.677	CZ: 75,5% ♀, 24,5% ♂;<br>SK: 63,9% ♀, 36,1%,<br>Studenten,	n. b.	1. Welle	AUDIT	Score gesamt 6,1±4,67 (0-30), CZ 6,1±4,51 (0-28), SK: 6,1±4,8 (0-30.<br>Alter für CZ neg. ass. mit Alkoholkonsum.
**Hanafi, 2021 [28]**	Indonesien	N=4.584<br>n=436 pos. A.	37,7% ♀, 60,3% ♂	30,4±6,8	04-06/20	AUDIT	Prävalenz 9%; Score 3,5 ± 4,7.<br>↑ bei 25,7%, ↔ bei 44,5%, ↓ bei 29,8% Ältere und Männer tranken mehr*

**Hennein, 2020 [29]**	USA	1.132	71,4% ♀, 28,3% ♂ medizinisches Personal	ab 18	05/20	AUDIT-C	42,6% mit Alkoholkonsumstörung, Score 4,8
**Huckle, 2020 [30]**	Neuseeland	2.132	78,6% ♀, 21,4% ♂	ab 18	04-05/20	n. s. F.	↑ bei 46,7%, ↔ bei 27,8%, ↓ bei 25,5%
**Jaffe, 2021 [31]**	USA	263	51,1% ♀, 38,8% ♂ Studenten	20,5±2,5	SS 2018 SS 2019 SS 2020	TLFB	↑ bei 16,5%, ↔ bei 43,3%, ↓ bei 40,5%
**Killgore, 2020 [33]**	USA	5.931	53,7% ♀, 46,3% ♂	18-84, 36,3±12,1	04-09/20	AUDIT	3.9 % in April → 17.4% in September in allen Gruppen (gefährlich, schädigend, abhängig)
**Koopmann, 2021 [34]**	Deutschland	3.245	63,9% ♀, 35,8% ♂	18->65	04-05/20	n. s. F.	↑ bei 35,5%, ↔ bei 42,9%, ↓ bei 21,3% Alter 25–34/35-44 mehr als andere Altersgruppen.
**Levy, 2021 [35]**	Israel	n1=477, n2=35	72,9% ♀, 27,1% ♂	n. b.	1.Welle (5/20); 2.Welle (10-11/20)	n. s. F.	Signifikante Unterschiede im Alkoholkonsum in Abhängigkeit von der COVID-19-Dauer. ♂>♀
**Manthey, 2021 [36]**	EU (D)	35.753	52,1% ♀, 47,2% ♂	> 18	04-07/20	AUDIT-C	Score 4,5; 17,8% mit Score > 8. ↑ bei 10 % Relevanter Anstieg der Häufigkeit des Alkoholkonsums für Deutschland und UK im Vergleich zu allen anderen Ländern).
**Martínez-Cao, 2021 [37]**	Spanien	21.207	69,9% ♀, 30,1% ♂	39,7±14,0	03/20	n. s. F.	13,5% berichteten über Alkoholkonsum als Copingstrategie
**McKetta, 2021 [38]**	USA	n1= 8.021 n2=20.038	n1:57,9% ♀, 42,1% ♂, n2: 57,6% ♀, 42,4 % ♂	n1: 52,2±15,7 n2: 52,8±16,1	03/20 und 05-06/20	n. s. F.	↑ der Trinktage im Verlauf der Pandemie***
**Minhas, 2021 [39]**	Canada	n1=730, n2=473	58,3% ♀, 41,7% ♂	23,8±1,2	2017 06-07/20	n. s. F.	↓ des starken Alkoholkonsums*, Tage mit starkem Alkoholkonsum**

**Mongeau-Pérusse, 2021 [40]**	Canada	847	77,8% ♀, 22,2% ♂ 42,5 % Gesundheits-personal		05-06/20	n. s. F.	↑ des täglichen Alkoholkonsums *** Gesundheitspersonal konsumierte weniger hochprozentige Alkoholprodukte während der Begrenzungen
**Oksanen, 2020 [42]**	Finnland	1.308 2019), 1.081 (2020	48,2% ♀, 51,8 ♂	19-65, 3,07±12,7	09-10/19; 03-04/20	AUDIT-C	↑ bei 25,4% (eher bei Jüngeren 30-45 Jahren), ↔ bei 48,02 %, ↓ bei 26,62 %
**Opara, 2021 [43]**	USA	575	55,6% ♀, 44,4% ♂	27,9±4,1	07-10/20	PACS	↑ bei 51,6% der ♂ und bei 48,4% der ♀. 23,4% mit positivem COVID-19-Diagnose, davon 47,2 % ↑ Alkoholkonsum und 16,7 % nicht zugenommen
**Price 2020 [45]**	Kanada	2.005	50% ♀ + ♂, Online-Spieler	18-24	04/20	n. s. F.	↑ bei 40,7%
**Ren, 2020 [47]**	China	1.172	69,3% ♀, 30,7% ♂	21,0–37,0, 22,0	02-03/20	n. s. F.	↑ bei 11,3%
**Rodriguez, 2020 [50]**	USA	754	50% ♀ + ♂	41,7±10,39	04/20	n. s. F.	13% ↑ Anzahl von Getränken, 16% ↑ mit schweren Trinktagen, ♀>♂
**Schecke, 2021 [52]**	Deutschland	2813	100% ♀	18 - >65	10-12/20	n. s. F.	↑ bei 23%, ↓ bei 18,5%
**Sidor, 2020 [53]**	Polen	1.097	95,1% ♀, 4,9% ♂,	18–71, 27,7±9,0	04-05/20	n. s. F.	↑ bei 14,6%, 8,3 % unsicher, ↔ bei 77 %
**Suffoletto, 2020 [54]**	USA	50	64% ♀, 36% ♂	18-25, 22,2±2,1	04/20	AUDIT-C	1. Woche 44% mit gefährlichem Konsum, nach 2. Woche 29% in 2. Woche, danach ↑ auf 65%
**Sun, 2020 [55]**	China	6.416	53% ♀, 47% ♂	28,23±9,23	03/20	n. s. F.	↑31,3% -> 32,7%; 18,7% rückfällig, 1,7% Beginn, ↓ bei 1,6 %
**Szajnoga, 2020 [56]**	Polen	4.072	84,4% ♀, 15,6 % ♂	11-77, 29,6 ± 9,4	04/20	n. s. F.	↑ bei 17,9%, ↔ bei 42,8%, ↓ bei 39,3 %

**Wardell, 2020 [57]**	Kanada	320	45,3% ♀, 54,7% ♂	32,0±9,2	04-05/2020	n. s. F.	↑ Häufigkeit und Intensität (*),<br>↑ alleinigen Trinkens
**Winkler, 2020 [60]**	Tschechien	3.306 (11/17)<br>3.021 (05/20)	54%♀, 46% ♂ (2017)<br>52% ♀, 48% ♂ (2020)	2017: 48,82 ± 17,19, 2020: 46,84 ± 16,02	2017<br>05/20	n. s. F.	↔ Missbrauch und Abhängigkeit,<br>↑ Binge-Drinking

Anmerkungen:

[t]aus Platzgründen werden nur die Erstautoren genannt; ♀= Frauen, ♂= Männer; OR = Odd Ratios, Ø = kein

AUDIT = Alcohol Use Disorder Identification Test, AUDIT-C = Alcohol Use Disorders Identification Test—Concise, TLFB = Timeline Follow-Back Interview, PACS = Penn Alcohol Craving Scale

* $p<0,05$, ** $p<0,01$ und *** $p<0,001$

n. b. = nicht berichtet, n. s. F = nicht standardisierter Fragebogen
